# Development of an adaptive, personalized, and scalable dementia care program: Early findings from the Care Ecosystem

**DOI:** 10.1371/journal.pmed.1002260

**Published:** 2017-03-21

**Authors:** Katherine L. Possin, Jennifer Merrilees, Stephen J. Bonasera, Alissa Bernstein, Winston Chiong, Kirby Lee, Leslie Wilson, Sarah M. Hooper, Sarah Dulaney, Tamara Braley, Sutep Laohavanich, Julie E. Feuer, Amy M. Clark, Michael W. Schaffer, A. Katrin Schenk, Julia Heunis, Paulina Ong, Kristen M. Cook, Angela D. Bowhay, Rosalie Gearhart, Anna Chodos, Georges Naasan, Andrew B. Bindman, Daniel Dohan, Christine Ritchie, Bruce L. Miller

**Affiliations:** 1 Department of Neurology, Memory and Aging Center, University of California, San Francisco, San Francisco, California, United States of America; 2 Global Brain Health Institute, University of California, San Francisco, San Francisco, California, United States of America; 3 Division of Geriatrics, Department of Internal Medicine, Home Instead Center for Successful Aging, Omaha, Nebraska, United States of America; 4 Philip R. Lee Institute for Health Policy Studies, University of California, San Francisco, San Francisco, California, United States of America; 5 Department of Medicine and Clinical Pharmacy, University of California, San Francisco, San Francisco, California, United States of America; 6 UCSF/UC Consortium on Law, Science & Health Policy, UC Hastings College of the Law, San Francisco, California, United States of America; 7 Department of Physics, Randolph College, Lynchburg, Virginia, United States of America; 8 Department of Pharmacy Practice, College of Pharmacy, University of Nebraska Medical Center, Nebraska Medical Center, Omaha, Nebraska, United States of America; 9 Division of General Internal Medicine, University of California, San Francisco/San Francisco General Hospital and Trauma Center, San Francisco, California, United States of America; 10 Division of Geriatrics, Department of Medicine, University of California, San Francisco, San Francisco, California, United States of America

## Abstract

Katherine Possin and colleagues report on the implementation, development, and early findings of the Care Ecosystem, an adaptive, personalized, and scalable dementia care program.

**Summary points**

- Our objective was to develop and test a scalable model of dementia specialty care that complements primary care with additional caregiver support and education, medication consultation, and support in planning for future medical, financial, and legal decisions consistent with patient values. Care is delivered via the phone and web by unlicensed Care Team Navigators (CTNs), who are trained and supervised by a dementia specialist nurse, social worker, and pharmacist.
- This “Care Ecosystem” is being tested via a pragmatic randomized controlled trial. The care model was iteratively improved during the trial based on input from caregivers, primary care providers, and clinical team members.
- Based on the inputs, the care model was revised to enhance caregiver support, to clarify triage protocols, to include more strategies for managing problematic patient behaviors, to personalize care protocols, to better inform caregivers and patients about the services available, to adjust recruitment efforts to target the underserved, and to address CTN stress and burnout.
- The trial continues with the revised care model and ongoing evaluation of patient, caregiver, and health care cost outcomes. New implementation projects are adapting the care model to fit the priorities and workflows of three health care delivery organizations.

## The challenge

The prevailing crisis management approach to dementia care does not adequately prepare and support patients and caregivers to manage the evolving challenges they face. Providers are reimbursed for brief problem-focused, in-person visits during which they simultaneously address multiple comorbid conditions [1]. Recent Centers for Medicare and Medicaid Services (CMS) policy changes provide reimbursement for monthly chronic care management by clinical staff that could provide an added layer of support [2]. A comprehensive care plan must be established, implemented, monitored, and revised as needed. Our challenge was to develop, improve, and test a new model of dementia care that addresses unmet needs of patients and caregivers, and aligns with the CMS’s initiatives for better health, better care, and lower costs [3].

## The Care Ecosystem model

**Fig 1:**
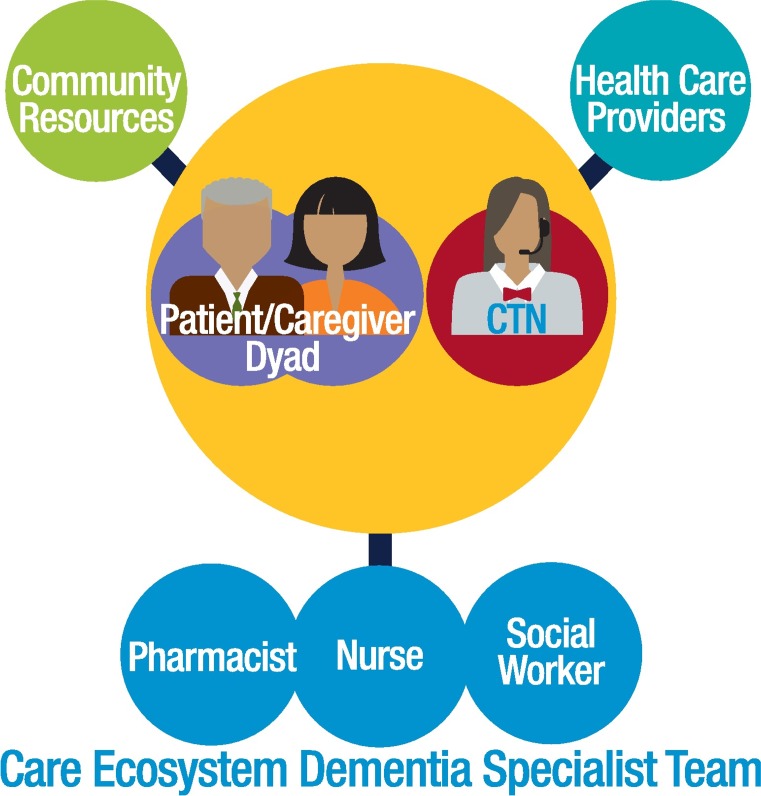
The Care Ecosystem model.

The Care Ecosystem is a telephone-based supportive care intervention for patients with dementia and their caregivers. The “Care Team Navigator” (CTN) is an unlicensed, trained dementia care guide who serves as the patient and caregiver’s primary point of contact. CTNs screen for common problems and provide support and standardized education. Contact frequency is scaled based on each patient and caregiver’s (dyad’s) needs and preferences but is typically monthy. CTNs triage complex medical or social issues to a nurse, social worker, and pharmacist, who have expertise in dementia care, and coordinate care with other health providers (Fig 1). CTNs are selected for strong communication and listening skills. They undergo 40 hours of didactic training through video or in-person lectures, assigned reading, and clinical observation. Ongoing training occurs during weekly case reviews and supervision (“clinical debriefings”) with the nurse, social worker, and pharmacist. Workflows and communication among the clinical team is supported by the Care Ecosystem Dashboard, a Salesforce software program developed with input from clinical staff.

The Care Ecosystem is informed by successful evidence-based programs that also include a nonlicensed dementia care role and emphasize caregiver support and education [4–7]. Unique to the Care Ecosystem is the extension of dementia specialty care to patients and caregivers for whom access to this care is limited, for reasons such as travel distance. Additionally, CTNs play a significant role in advance care planning discussions, medication reconciliation, and monitoring health status. Their role is to identify needs, provide standardized education, and prepare dyads for appointments with licensed providers.

## The Care Ecosystem trial

**Fig 2:**
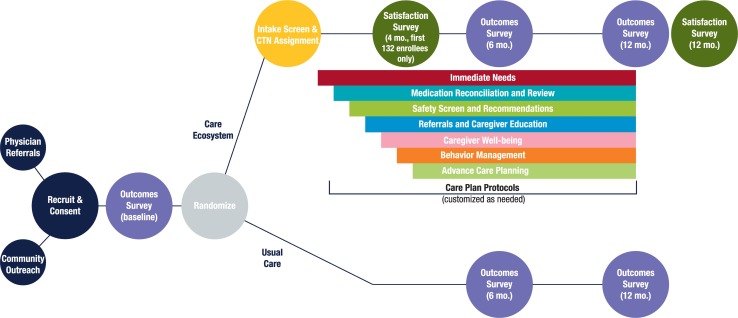
The Care Ecosystem trial.

The impact of the Care Ecosystem on patient, caregiver, and cost outcomes is being tested through a pragmatic, randomized controlled trial [8] funded by a Centers for Medicare & Medicaid Innovation (CMMI) Healthcare Innovations Award Round Two (September 1, 2014, to August 31, 2017; see Fig 2). Inclusion criteria are dementia diagnosis of any type by any medical provider; age ≥45; Medicare- or Medicaid-enrolled or pending; California, Nebraska, or Iowa residence; a caregiver who may or may not reside with the patient; and fluency in English, Spanish, or Cantonese. Exclusion criteria are persons with a life expectancy of less than 3 months or living in a nursing home at time of enrollment. Consent materials were approved by the University of California San Francisco (UCSF) and the University of Nebraska Medical Center (UNMC) Institutional Review Boards. Patients with capacity for written informed consent are given the opportunity to consent for themselves; otherwise, consent is obtained from a legally authorized representative. Primary caregivers provide written consent. Dyads are randomly assigned to the Care Ecosystem intervention or usual care at a 2:1 ratio. The intervention continues for 1 year or until trial end. Target enrollment is 1,050 dyads. Baseline characteristics of dyads enrolled are presented in Table 1.

**Table 1:** Characteristics of persons with dementia and caregivers enrolled as of September 1, 2016.

A. Persons with Dementia			B. Caregivers		
	Care Ecosystem	Usual Care		Care Ecosystem	Usual Care
***n***	400	210	***n***	400	210
**Age** M(SD)	78.8 (8.7)	77.3 (10.1)	**Age** M(SD)	66.8 (11.7)	65.5 (11.2)
**Ethnicity** (%)			**Ethnicity** (%)		
Caucasian	82.0	84.8	Caucasian	81.5	85.2
Asian	5.5	4.8	Asian	6.5	4.3
Black or African American	4.5	3.3	Black or African American	4.5	2.9
Other	8.0	7.1	Other	7.5	7.6
**Education** (%)			**Education** (%)		
<12 years	7.0	6.2	<12 years	1.8	1.4
12 years	20.3	22.4	12 years	7.3	13.3
13–15 years	21.3	18.1	13–15 years	25.6	20.5
≥16 years	51.5	53.3	≥16 years	65.4	64.8
**Female** (%)	58.3	51.0	**Female** (%)	67.2	69.5
**State of residence** (%)			**Caregiver relationship** (%)		
California	56.8	53.8	Wife	34.5	41.0
Nebraska	38.3	38.1	Daughter	26.8	24.3
Iowa	5.0	8.1	Husband	24.8	20.5
**Annual household income** (%)			Son	6.3	8.1
<$15,000	2.6	3.0	Sibling or Other	7.7	6.2
$15,000–$49,999	26.0	23.1	**Severe caregiver burden** (%)	50.5	53.3
$50,000–$99,999	31.4	32.0	**Mild–severe depression** (%)	39.5	41.0
$100,000–$149,999	16.7	19.5			
≥$150,000	14.4	12.4			
Don’t know/refused	9.0	10.1			
**Stage**					
Mild	49.8	50.5			
Moderate	30.8	31.4			
Advanced	19.5	18.1			
**Comorbidities**					
Cardiovascular	54.5	45.7			
Arthritis/rheumatism	43.8	41.9			
Depression	42.8	39.5			
Stroke	15.0	11.4			
Diabetes	13.5	13.3			
**QoL-AD** Mean (SD)	32.9 (5.8)	33.4 (5.8)			
**Emergency care utilization,** last 6 months					
Hospitalization rate	0.31	0.27			
ER visit rate	0.37	0.47			

Characteristics were collected during the baseline Outcomes Survey with the caregivers. Stage of dementia was determined using the Quick Dementia Rating Scale using published cut-offs that correspond to the Clinical Dementia Rating Scale [9]. The most common comorbidities are reported based on questions from the Charleston Comorbidity questionnaire [10,11]. Hospitalization and ER visit rates are the number of visits in the prior 6 months divided by the number of patients. On the Qol-AD, caregivers rate patient quality of life on 13 items as poor, fair, good, or excellent, and scores can range from 13–52 [12]. Ratings that are “fair” on average result in a score of 26 and “good” in a score of 39. Caregiver burden is based on the Zarit-12, with scores >17 indicating severe [13]. Caregiver depression is based on the PhQ-9, with scores >4 indicating mild depression [14].

ER, emergency room; M, mean; QoL-AD, quality of life in Alzheimer disease; SD, standard deviation.

Prior to starting the trial, we worked with our respective Institutional Review Boards to ensure that appropriate human subject protections were in place. We review any incidents or adverse events with our Data Safety Monitoring Board and the UCSF Privacy Office. To date, we have had three reportable incidents, all involving a breach of privacy, in which a staff person accidentally sent confidential information to the wrong participant. We instituted a peer check protocol to prevent future incidents.

### Trial outcomes

A 30-minute Outcome Survey is administred by phone prior to randomization and at 6 and 12 months post-randomization by a research coordinator blinded to randomization status (S1 Table). Medicare and Medicaid claims data are analyzed to evaluate cost of care. At the end of the 12-month outcomes survey, the coordinator is instantly unblinded so that they can administer a 10-minute Satisfaction Survey to caregivers in the intervention group before ending the call (see S1 Text). This Satisfaction Survey was also administered to the first 132 caregivers randomized to the intervention, 4 months post-randomization, to support agile development of the care model (see below).

### Agile development of the care model

**Fig 3:**
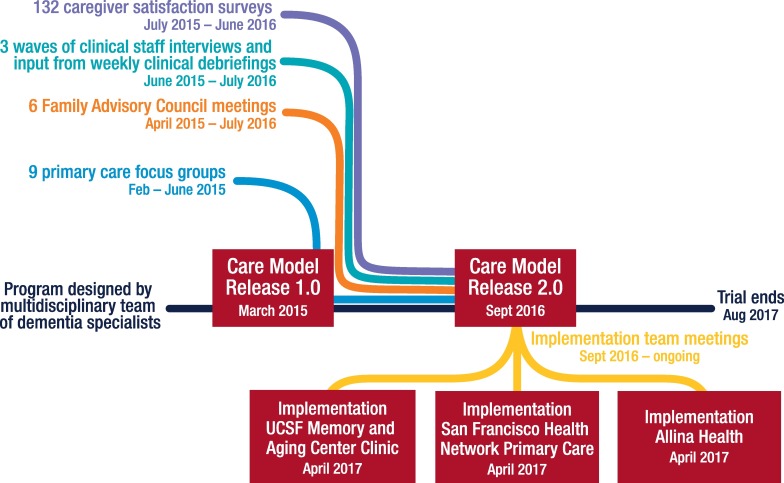
Agile development of the care model.

Without compromising standardization of core program elements, including the composition of the clinical team and our emphases on caregiver support and education, medications, and advance care planning, we applied “agile” software development principles [15,16] to refine the care model based on user input. These principles, as applied to our care model development, were a care model that is adapted efficiently based on user inputs, modular care protocols with well-defined integration points, collaborative development and revisions by a multidisciplinary team, incremental revisions with minor releases (approximately monthly) and major releases (Care Model 1.0, Care Model 2.0, and the three implementation releases), and users broadly defined to include caregivers, clinical team members, and primary care providers. Feedback was used to revise modular care protocols, CTN training, patient and caregiver educational materials, and our Care Ecosystem Dashboard. These revisions did not require consent form modifications. The user inputs included 4-month Caregiver Satisfaction Surveys, quarterly meetings with the UCSF Memory and Aging Center Family Advisory Council comprising 13 dementia family caregivers, three phases of interviews with all Care Ecosystem clinical staff and input gathered during weekly clinical debriefings, and nine primary care provider focus groups. The provider focus groups, conducted between February and June 2015, were led by a geriatrician, included a total of 43 physicians and 6 nurse practitioners, and asked about perspectives on care in dementia, preferred methods of care coordination, and their reaction to the Care Ecosystem model. Qualitiative data analysis of user inputs guided program revisions, which were complete by September 2016, creating Care Model 2.0. See Fig 3.

### Trial status

As of September 1, 2016, when Care Model 2.0 was released, 610 dyads were enrolled in the Care Ecosystem trial, with 400 randomized to treatment and 210 to usual care (Table 1). Enrollment continued through February 28, 2017. A UCSF clinical team provides services to 227 California residents, and an independent UNMC clinical team provides services to 153 Nebraska and 20 Iowa residents (see S1 Fig for locations of enrolled patients). The current maximum caseload for a CTN is 64, and the target maximum caseload is 80. Bilingual CTNs provide care in the recipient’s preferred language (English, Spanish, or Cantonese).

### Caregiver satisfaction ratings

Satisfaction ratings were obtained during the 4-month Caregiver Satisfaction Survey. Ninety-two percent would recommend the Care Ecosystem to other caregivers. Seventy-seven percent said they needed help from their CTN at some point since enrollment, and 96% of these said the CTN was available when needed. Ninety-two percent said the amount of contact they had with their CTN was about right, 7% said that there was too little contact, and none said they had too much contact (1% did not respond). Fifty-nine percent reported that the CTN referred them to resources in their community, and 87% of these said the referrals were helpful. Forty-nine percent of caregivers indicated that the patient had problematic behavioral changes; of these, 65% said the CTN helped them manage those behaviors. Eleven percent of caregivers indicated that they thought the Care Ecosystem helped the patient avoid an unnecessary emergency room or hospital visit. Responses to open-ended questions on the Satisfaction Survey were analyzed using Atlas.Ti, a qualitative data coding software.

## Care model 2.0: User input and program revisions

### Increase emphasis on caregiver support

Caregivers, clinical staff, and primary care providers all highlighted the importance of caregiver support for reducing caregiver distress. This was emphasized in seven of the nine primary care focus groups and in all six meetings with the Family Advisory Council. On the Satisfaction Survey, 83 caregivers commented favorably on aspects of the Care Ecosystem intervention that focused on caregiver support, such as the provision of information and resouces, referrals to respite programs, and general emotional support. Of these caregivers, 35 specifically referred to the benefits of the relationship with their particular CTN.

To enhance this support, the CTN training curriculum was updated to improve competency in balancing rapport building with direct problem-solving, and sample scripts were created to help guide conversations around difficult topics regularly raised by dementia caregivers. Open-ended questions are now employed in every call to identify needs. Additionally, CTNs were trained to identify and proactively find solutions to problems impacting caregiver well-being (e.g., inattention to personal health, need for respite, loneliness, and grief).

### Clearer triage protocols

Our licensed clinical staff emphasized the need to clarify scope of each role on the team for best practices. On the Satisfaction Survey, 12 caregivers commented on deficits in CTN knowledge or abilities. Providers in six focus groups expressed that while they appreciated that CTNs could help identify health issues between visits and prepare families for provider appointments, all medical guidance must be deferred to licensed clinicians. This feedback highlighted the limits of using a nonlicensed staff member as the first point of contact in delivering specialty care. We clarified training protocols around when and how to triage to the Care Ecosystem nurse, social worker, and pharmacist, and to the patient’s primary care provider, as follows.

### Advance care planning

The role of the CTN was limited to include identifying gaps in planning (e.g., identifying a health care agent), making legal referrals, and preparing the family for a billable advance care planning appointment with their primary care provider [17].

### Monitoring health status

CTNs provide education and screen monthly for acute health changes that could signal delirium. Concerns identified by the CTNs are triaged to the nurse immediately.

### Managing challenging behaviors

CTNs screen for challenging behaviors during the Intake Screen, listen for behavior problems mentioned by the caregiver at any time, and provide targeted educational handouts. If the situation does not improve or the caregiver is significantly distressed, the nurse consults directly with the caregiver.

### Medication management

The CTNs reconcile what the patient is taking and check in monthly for possible side effects or adherence problems. The pharmacist provides medication reviews and recommendations to the prescribing providers and caregivers.

### Personalization of protocol

On the Satisfaction Survey, 45 caregivers indicated that our medication and advance care planning protocols were duplicative with work they had done elsewhere, and CTNs noted that these structured protocols could interfere with building rapport. Now each protocol starts with a needs assessment and the CTNs address only identified needs.

### Creation of service menu

Twelve caregivers expressed a lack of clarity around the extent of services the Care Ecosystem offered. Therefore, now we include a Care Ecosystem service list in our welcome packet, and the CTN refers to this list in communications with the dyad.

### Adjustment of recruitment efforts

Initially, the most efficient recruitment pipelines were an academic medical center tertiary care clinic (the UCSF Memory and Aging Center Clinic) and self-referrals. This resulted in a disproportionately white, high-income, and English-speaking sample (see Table 1). Primary care providers in five of the focus groups highlighted care gaps for the underserved, and some of our high-income caregivers indicated that their needs were already well met. Now, we emphasize recruitment of low-income, ethnically diverse, monolingual Chinese and Spanish speakers, and rural-dwelling persons. Currently, we suspect that the benefit experienced by the underserved will be greater.

### Addressing CTN stress and burnout

CTNs expressed that listening to patients and caregivers talk about grief, loss, and frustration carries an emotional toll. We now provide a monthly workshop facilitated by a mental health specialist or chaplain to provide CTNs with emotional support and coping strategies.

## Next steps and future directions

We have initiated implementation projects with Allina Health primary care clinics in Minnesota, the San Francisco Health Network primary care clinics, and the UCSF Memory and Aging Center dementia specialty clinic. The Care Ecosystem will be integrated into these clinics with on-site CTNs who have direct access to electronic medical records, facilitating a level of care coordination that was not possible during the trial. Without the burdens of research measurement and randomization to a control group, we anticipate that the program will be more appealing to distressed caregivers. Also, at the San Francisco Health Network clinics, CTNs will help address a major barrier to care—late or missed diagnosis—by providing cognitive and functional screens when they are requested by the primary care provider. The three implementation projects are beginning with regular meetings to carefully understand each site’s workflows and priority improvement opportunities to guide adaptations of Care Model 2.0 that are specific for each health care delivery system [18]. Concurrently with these implementation projects, we hope to measure the long-term impacts of Care Ecosystem in the CMMI trial cohort.

The iterative design of our trial allowed us to improve the care model based on our learnings but will complicate the interpretation of treatment effects. Importantly, the core program elements remained standard to permit analyses in the full sample. Secondary analyses that include a time-varying covariate will help us understand and communicate the impact of the revisions on study outcomes.

## Supporting information

S1 Table Outcome Survey measures. (DOCX)

S1 Text The Caregiver Satisfaction Survey. (DOCX)

S1 Fig Zip codes in which Care Ecosystem participants with dementia are enrolled. (TIF)

## Abbreviations

CMMI: Centers for Medicare & Medicaid Innovation
CMS: Centers for Medicare and Medicaid Services
CTN: Care Team Navigator
UCSF: University of California San Francisco
UNMC: University of Nebraska Medical Center

## References

[1] Hinton L, Franz CE, Reddy G, Flores Y, Kravitz RL, Barker JC. Practice constraints, behavioral problems, and dementia care: primary care physicians' perspectives. J Gen Intern Med. 2007;22(11):1487–92. PubMed Central PMCID: PMCPMC2219799. 10.1007/s11606-007-0317-y 17823840 PMC2219799

[2] Medicare Learning Network: Chronic Care Management Services: Department of Health and Human Services; 2016 [cited 2017 January 23]. https://www.cms.gov/Outreach-and-Education/Medicare-Learning-Network-MLN/MLNProducts/Downloads/ChronicCareManagement.pdf.

[3] Strengthening Medicare: Better Health, Better Care, Lower Costs, Efforts Will Save Nearly $120 Billion for Medicare Over Five Years: Centers for Medicare and Medicaid Services; [cited 2017 January 23]. https://www.cms.gov/apps/files/medicare-savings-report.pdf.

[4] Goeman D, Renehan E, Koch S. What is the effectiveness of the support worker role for people with dementia and their carers? A systematic review. BMC Health Serv Res. 2016;16:285 PubMed Central PMCID: PMCPMC4950786. 10.1186/s12913-016-1531-2 27435089 PMC4950786

[5] LaMantia MA, Alder CA, Callahan CM, Gao S, French DD, Austrom MG, et al The Aging Brain Care Medical Home: Preliminary Data. J Am Geriatr Soc. 2015;63(6):1209–13. 10.1111/jgs.13447 26096394

[6] Mittelman MS, Brodaty H, Wallen AS, Burns A. A three-country randomized controlled trial of a psychosocial intervention for caregivers combined with pharmacological treatment for patients with Alzheimer disease: effects on caregiver depression. Am J Geriatr Psychiatry. 2008;16(11):893–904. PubMed Central PMCID: PMCPMC2753499. 10.1097/JGP.0b013e3181898095 18978250 PMC2753499

[7] Reuben DB, Evertson LC, Wenger NS, Serrano K, Chodosh J, Ercoli L, et al The University of California at Los Angeles Alzheimer's and Dementia Care program for comprehensive, coordinated, patient-centered care: preliminary data. J Am Geriatr Soc. 2013;61(12):2214–8. PubMed Central PMCID: PMCPMC3889469. 10.1111/jgs.12562 24329821 PMC3889469

[8] Thorpe KE, Zwarenstein M, Oxman AD, Treweek S, Furberg CD, Altman DG, et al A pragmatic-explanatory continuum indicator summary (PRECIS): a tool to help trial designers. J Clin Epidemiol. 2009;62(5):464–75. 10.1016/j.jclinepi.2008.12.011 19348971

[9] Galvin JE. The Quick Dementia Rating System (Qdrs): A Rapid Dementia Staging Tool. Alzheimers Dement (Amst). 2015;1(2):249–59. PubMed Central PMCID: PMCPMC4484882. 26140284 10.1016/j.dadm.2015.03.003 PMC4484882

[10] Charlson ME, Pompei P, Ales KL, MacKenzie CR. A new method of classifying prognostic comorbidity in longitudinal studies: development and validation. J Chronic Dis. 1987;40(5):373–83. 3558716 10.1016/0021-9681(87)90171-8

[11] Chaudhry S, Jin L, Meltzer D. Use of a self-report-generated Charlson Comorbidity Index for predicting mortality. Med Care. 2005;43(6):607–15. 15908856 10.1097/01.mlr.0000163658.65008.ec

[12] Logsdon RG, Gibbons LE, McCurry SM, Teri L. Assessing quality of life in older adults with cognitive impairment. Psychosom Med. 2002;64(3):510–9. 12021425 10.1097/00006842-200205000-00016

[13] Zarit SH, Reever KE, Bach-Peterson J. Relatives of the impaired elderly: correlates of feelings of burden. Gerontologist. 1980;20(6):649–55. 7203086 10.1093/geront/20.6.649

[14] Kroenke K, Spitzer RL, Williams JB. The PHQ-15: validity of a new measure for evaluating the severity of somatic symptoms. Psychosom Med. 2002;64(2):258–66. 11914441 10.1097/00006842-200203000-00008

[15] Cohen D, Lindvall, M., & Costa, P. An Introduction to Agile Methods. Advances in Computers [Internet]. 2004 [cited 2016 Sep 29]; 62. http://www.cse.chalmers.se/~feldt/courses/agile/cohen_2004_intro_to_agile_methods.pdf.

[16] Abrahamsson P, Salo O., Ronkainen J., & Warsta J. Agile software development methods: Review and analysis. VTT Publications [Internet]. 2002 [cited 2016 Sep 29]; 478. http://www.vtt.fi/inf/pdf/publications/2002/P478.pdf.

[17] Medicare Learning Network: Advance Care Planning: Department of Health and Human Services; 2016 [cited 2017 January 23]. https://www.cms.gov/Outreach-and-Education/Medicare-Learning-Network-MLN/MLNProducts/Downloads/AdvanceCarePlanning.pdf.

[18] Boustani MA, Munger S, Gulati R, Vogel M, Beck RA, Callahan CM. Selecting a change and evaluating its impact on the performance of a complex adaptive health care delivery system. Clin Interv Aging. 2010;5:141–8. PubMed Central PMCID: PMCPMC2877524. 20517483 10.2147/cia.s9922 PMC2877524

