# Supplementary material for: Development of an adaptive, personalized, and scalable dementia care program: Early findings from the Care Ecosystem
Source: PLoS Med. 2017 Mar 21;14(3):e1002260. doi: 10.1371/journal.pmed.1002260 (PMC5360211; doi:10.1371/journal.pmed.1002260)
Supplement: S1 Table — (DOCX) [file pmed.1002260.s001.docx]

**S1 Table. Outcome Survey Measures**.

*Administered to the caregiver*

Caregiver burden (Zarit-12) [1]

Caregiver depression (PHQ-9) [2]

Caregiver self-efficacy (four items)

Patient quality of life (QoL-AD) [3]

Dementia staging (QDRS) [4]

Functional status (OASIS)[5]

Patient health care utilization, with questions adapted from the Health and Retirement Survey [6]

Patient and caregiver demographic characteristics

*Administered to patients who are able to complete a phone survey*

Patient quality of life (QoL-AD) [3]

Cognitive screen (NINDS-5 minute screen)[7]

**References:**

1. Zarit SH, Reever KE, Bach-Peterson J. Relatives of the impaired elderly: correlates of feelings of burden. Gerontologist. 1980;20(6):649-55. PubMed PMID: 7203086.

2. Kroenke K, Spitzer RL, Williams JB. The PHQ-15: validity of a new measure for evaluating the severity of somatic symptoms. Psychosom Med. 2002;64(2):258-66. PubMed PMID: 11914441.

3. Logsdon RG, Gibbons LE, McCurry SM, Teri L. Assessing quality of life in older adults with cognitive impairment. Psychosom Med. 2002;64(3):510-9. PubMed PMID: 12021425.

4. Galvin JE. The Quick Dementia Rating System (Qdrs): A Rapid Dementia Staging Tool. Alzheimers Dement (Amst). 2015;1(2):249-59. doi: 10.1016/j.dadm.2015.03.003. PubMed PMID: 26140284; PubMed Central PMCID: PMCPMC4484882.

5. Fortinsky RH, Garcia RI, Joseph Sheehan T, Madigan EA, Tullai-McGuinness S. Measuring disability in Medicare home care patients: application of Rasch modeling to the outcome and assessment information set. Med Care. 2003;41(5):601-15. doi: 10.1097/01.MLR.0000062553.63745.7A. PubMed PMID: 12719685.

6. Hurd MD, Martorell P, Delavande A, Mullen KJ, Langa KM. Monetary costs of dementia in the United States. N Engl J Med. 2013;368(14):1326-34. doi: 10.1056/NEJMsa1204629. PubMed PMID: 23550670; PubMed Central PMCID: PMCPMC3959992.

7. Wong A, Nyenhuis D, Black SE, Law LS, Lo ES, Kwan PW, et al. Montreal Cognitive Assessment 5-minute protocol is a brief, valid, reliable, and feasible cognitive screen for telephone administration. Stroke. 2015;46(4):1059-64. doi: 10.1161/STROKEAHA.114.007253. PubMed PMID: 25700290; PubMed Central PMCID: PMCPMC4373962.
