# Supplementary material for: Development of an adaptive, personalized, and scalable dementia care program: Early findings from the Care Ecosystem
Source: PLoS Med. 2017 Mar 21;14(3):e1002260. doi: 10.1371/journal.pmed.1002260 (PMC5360211; doi:10.1371/journal.pmed.1002260)
Supplement: S1 Text — (DOCX) [file pmed.1002260.s002.docx]

**S1 Text. The Caregiver Satisfaction Survey**.

**What do you find most helpful about Care Ecosystem? How could we be more helpful?**

**Has (CTN) been available when you need him/her?**

- Yes
- No
- N/A (hasn't had to call)

If no, **please explain.**

**Would you say that the amount of contact you have had with (CTN) has been about right?**

- Yes
- Too Little Time
- Too Much Time

If TOO LITTLE or TOO MUCH: **Could you explain?**

**Has (CTN) referred you to resources in your community?**

- Yes
- No
- N/A (e.g. "I don't need them")

If “Yes” is selected: **Have these referrals been helpful? Please explain.**

- Yes
- No

**Has (CTN) helped you manage any behavioral changes associated with (patient)’s dementia (for example irritability, or lack of interest in doing things)?**

IF YES, ask: "In what ways?"

IF NO, ask: "How could (CTN) be more helpful?"

- Yes
- No (Patient has problematic behavioral changes but Care Ecosystem has not helped them)
- N/A (Patient does not have problematic behavioral changes)

**Do you think the Care Ecosystem helped you to avoid an emergency room or hospital visit for (patient)?**

IF YES, say "Please explain."

- Yes
- No

**Would you recommend Care Ecosystem to other caregivers?**

- Yes
- No

**When we share your answers to this survey with our team, is it OK if we include your name? Feel free to say no if you want your answers to remain anonymous.**

- Yes
- No
