# Supplementary figures and images for: Development of an adaptive, personalized, and scalable dementia care program: Early findings from the Care Ecosystem
Source: PLoS Med. 2017 Mar 21;14(3):e1002260. doi: 10.1371/journal.pmed.1002260 (PMC5360211; doi:10.1371/journal.pmed.1002260)

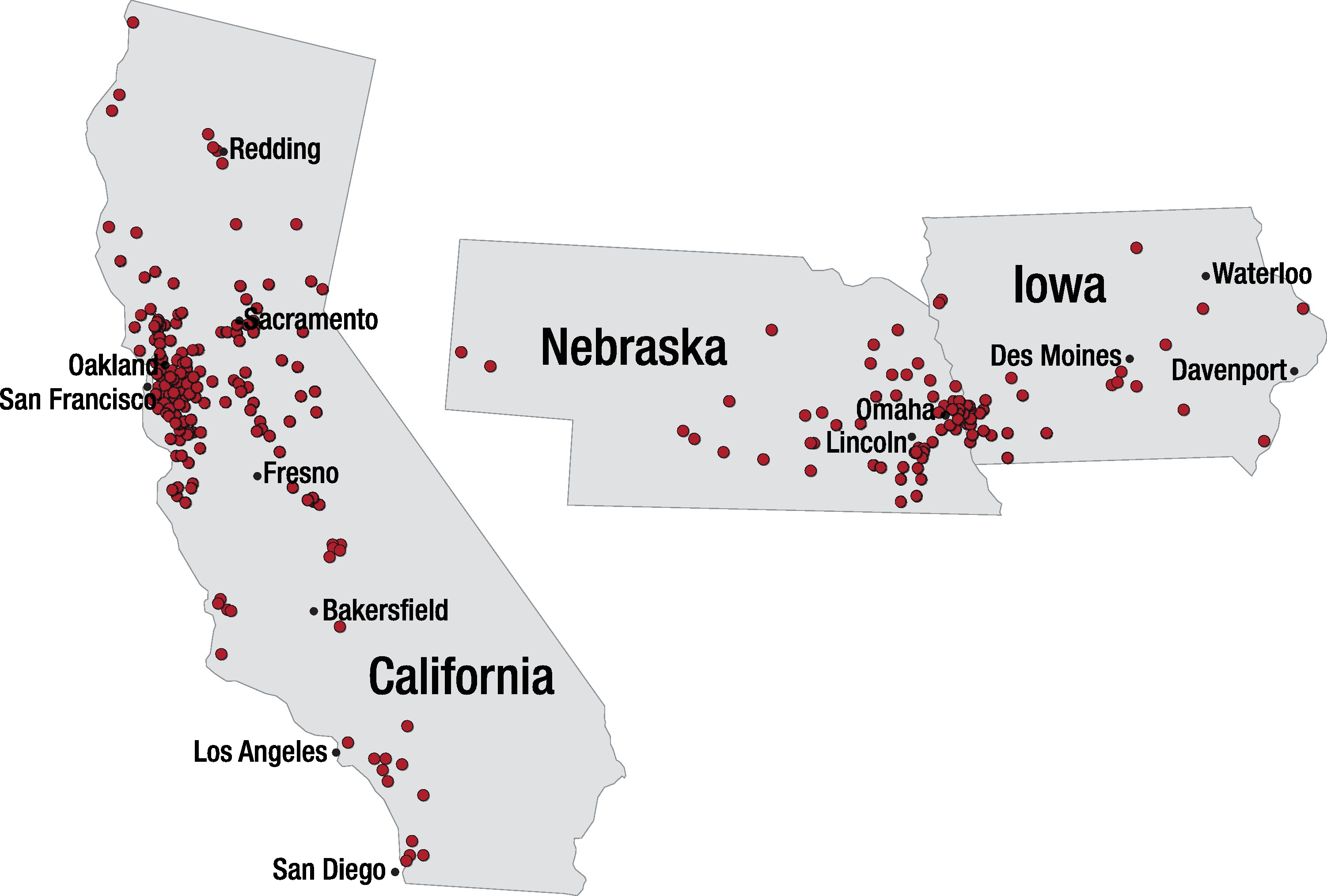

Supplement: S1 Fig — (TIF) [file pmed.1002260.s003.tif]
